# Identification of recurrent BRCA1 mutation and its clinical relevance in Chinese Triple‐negative breast cancer cohort

**DOI:** 10.1002/cam4.1004

**Published:** 2017-01-30

**Authors:** Xiaoran Liu, Huiping Li, Bin Shao, Jianmin Wu, Weiyao Kong, Guohong Song, Hanfang Jiang, Jing Wang, Fengling Wan

**Affiliations:** ^1^ Key laboratory of Carcinogenesis and Translational Research (Ministry of Education)Department of breast oncologyPeking University Cancer Hospital & InstituteFucheng Road No.52Haidian DistrictBeijing100142China

**Keywords:** BRCA, breast cancer, Chinese, epidemiology

## Abstract

Triple‐negative breast cancer (TNBC) accounts for 15–20% of all newly diagnosed breast cancers, and is enriched for germline mutation of *BRCA*. In Asian patients diagnosed with breast cancer, 268 deleterious mutations of *BRCA1* and 242 of *BRCA2* have been identified so far, including a reported *BRCA1* frameshift mutation (rs80350973), apparently found only in Asian people, with a low prevalence of 0.3–1.7% in different breast cancer cohorts. Here, we reported the high prevalence (7.2%) of rs80350973 among 125 Chinese patients with TNBC, which implies its mutational predilection for certain breast cancer subtypes. Although its low prevalence had not indicated any particular clinical significance in previous studies, our results associated rs80350973 mutation with cell checkpoint malfunction, and was found to be more common in TNBC patients with high Ki‐67 indices (*P *=* *0.004). As Ki‐67 overexpression is a predictor of poor prognosis in TNBC, inclusion of this mutation into genetic assessments may improve the clinical management of Chinese patients with TNBC.

## Introduction

Triple‐negative breast cancer (TNBC) is defined by little or no expression of estrogen receptor (ER), progesterone receptor (PR), and human epidermal growth factor receptor 2 (HER2). This clinical subtype accounts for 15–20% of all newly diagnosed breast cancers (BCs) 1. TNBCs possess highly unstable genomes; they are frequently diagnosed in young women and have a worse prognosis than other BC subtypes 2, 3. Furthermore, no promising regimen has so far been found for TNBC. Conventional chemotherapy remains the one effective treatment modality, but its long‐term clinical outcomes are unsatisfactory 4, 5. Earlier studies of germline *BRCA1* mutations in TNBC have shown wide variation in their prevalence, with a range of 10–40%. These mutations are associated with family history 6, 7, risk of recurrence 8, 9, and sensitivity to DNA‐damaging agents 10, 17.

Among Asians, 268 deleterious mutations of *BRCA1* and 242 of *BRCA2* so far have been documented in patients diagnosed with BC 12. Several studies of Chinese patients with TNBC found that the prevalence of *BRCA1* germline mutations varied from 18.6% to 36.8% 12. The high frequency of certain mutations in *BRCA1/2* has been widely studied, to optimize genetic testing strategies for those at high risk for BC. On the other hand, clinical trials suggest that *BRCA1/2* mutations in TNBCs could respond better to platinum‐based chemotherapy or other DNA‐damaging therapies 13, 14. Notably, current studies mostly focus on identifying novel mutations of *BRCA1/2* and their prevalence in TNBC. However, little is known about the high prevalence of certain *BRCA1/2* mutations and their clinical relevance in TNBC 12. In this study, we screened recurrent germline mutations, mainly against the *BRCA1* gene, and sought their clinical relevance in Chinese patients with TNBCs. The results of our work may help to interpret the clinical significance of certain recurrent mutations of *BRCA1*.

## Material and Methods

### Study cohort

A total 125 TNBC of 1300 newly diagnosed BC patients were recruited at our hospital, between January 2013 and June 2015. All subjects gave written informed consent. The inclusion criteria were as follows: (1) patients whose disease was pathologically shown to be negative for ER, PR, and HER2/ErbB2, and (2) for whom complete clinical, pathological and follow‐up data were available. The exclusion criteria were as follows: (1) patients without complete follow‐up data, and (2) those who suffered nontumor‐mediated death. Clinical data were obtained from review of medical records and patient interviews by clinical physicians. This study was approved by the medical ethics committee of our hospital. We initially performed whole‐exon mutations screening of 26 patients with TNBC, using panel‐based next‐generation sequencing (NGS) analysis of 55 susceptibility genes; frequencies of some recurrent mutations were later detected in the larger TNBC cohort using mutation site targeting PCR amplification. Amplified products were submitted to Sanger sequencing.

### Panel‐based NGS analysis

The first step for NGS technology was use of the TruSeq Custom Amplicon method to design oligo probes that are specific for all coding sequences and intron/exon boundaries of coding exons from the 55 genes that affect BC susceptibility (Table S1), using Illumina Design‐Studio (Illumina, Inc., San Diego, CA). For each 150‐bp sequence of the target region, a pair of oligo probes were synthesized to hybridize with the 5′ and 3′ ends of the sequence at one end (the other end was complementary to the PCR primers). These oligo probes were used to construct a library containing the necessary nucleotide sequences. The target regions were determined by selecting all exons of the 55 susceptibility genes; however, to include sections of the intron‐exon regions, the regions also included 50 nucleotides upstream and downstream of each exon.

Sequencing was performed using the NGS MiSeq Illumina sequencer (Illumina, Inc.). Obtained sequences were aligned to the reference genome (GRCh37/hg19) using MiSeq Reporter software (Illumina, Inc.), which detected discrepancies determining their type, such as deletions, insertions and SNPs. The sequences were analyzed using MiSeq software. As an acceptance threshold value, we selected a Q‐score of 30, which corresponds to a 1: 1000 error rate.

### Genotyping

Since rs80350973 is a heterozygous mutation, we design a pair of primers to amplify the germline DNA fragment containing this mutational site; unaligned sequences from the deletion site cause “double peaks” in Sanger sequencing, whereas the wild‐type will not.

In brief, DNA was extracted from peripheral blood using the QIAamp DNA Blood Mini Kit^®^ (Qiagen, Germantown, MD). Samples were submitted to PCR amplification targeting for *BRCA1* mutation (rs80350973). The PCR reaction used SYBR^®^ Green Realtime PCR Master Mix (Toyobo Co., Ltd, Kita‐ku, Osaka, JAPAN). Primers against rs80350973 mutation were as follows: forward primer 5′‐AGGACCCTGGAGTCGATTGA‐3′, reverse primer 5′‐GTAAGCTCATTCTTGGGGTCCTGT‐3′. Amplified products were submitted to sequencing in an ABI 3730 automated sequencer using the Big Dye Terminator v3.1 Cycle Sequencing Kit (Applied Biosystems, Foster City, CA) as described by the manufacturer. All analyses were performed in duplicate. Discrepancies in Sanger sequencing outcome between the wild type and rs80350973 mutation are shown in Figure S1.

### Immunohistochemistry of Ki‐67

In this study, Ki‐67 expression was quantified by a visual grading system and was determined by counting 1,000 tumor cells using the Olympus Image Analyzer (magnification 400×). Ki‐67 immunoreactivity (Ki‐67 index) was recorded as a continuous variable based on the proportion of positive tumor cells (0–100%), regardless of staining intensity. All cases were histopathologically confirmed independently by two experienced pathologists according to ASCO/CAP 2010 criteria.

### Statistical analysis

The statistical significance of associations between the *BRCA1* frameshift mutation and clinicopathological features of TNBC patients were evaluated using Pearson's *χ*2 tests or Fisher's exact tests as appropriate. The Mann–Whitney test was used to analyze associations between the Ki‐67 index and *BRCA1* frameshift mutations. Disease‐free survival (DFS) was defined as the time from the date of diagnosis to first recurrence (not including second primary malignancies) or death from BC without a recorded relapse. Lengths of DFS for patients with different axillary lymph node status (N0–2 vs. N3), and Ki‐67 index levels (≤0.7 vs.>0.7, and ≤0.14 vs. >0.14) were plotted with Kaplan–Meier curves and compared with log‐rank tests. DFS was compared between rs80350973 mutation carriers and noncarriers using a time‐dependent covariate Cox regression model, which took lymph node status (N0–2 vs. N3) and Ki‐67 indices into consideration. All statistical tests were two‐sided; *P *<* *0.05 was considered significant. Data analyses were calculated using SPSS^®^ 19.0 software (SPSS Inc., Chicago, IL).

## Results

### Patient characteristics

Clinical data of the 125 included TNBC patients are summarized in Table 1. Their median age at diagnosis was 47 years (range: 26–75 years). Of these 125 patients, 16 (12.8%) were diagnosed when they were younger than 35 years; 11 (8.8%) had family histories of breast or ovarian cancer. Invasive ductal carcinoma was common (90.4%). Their main metastasis sites were brain (*n *=* *11; 8.8%), lung (*n *=* *49; 39.2%), and liver (*n *=* *18; 14.4%).

**Table 1:** Clinicopathological characteristics of 125 Triple‐negative breast cancer patients

Characteristic	*n* (%)
Age at diagnosis (years)	
>35	109 (87.2)
≤35	16 (12.8)
Histology of primary tumor	
Ductal	113 (90.4)
Lobular	3 (2.4)
Others	7 (5.6)
Unknown	2 (1.6)
Primary tumor size	
T1	45 (36.0)
T2	51 (40.8)
T3	10 (8.0)
T4	8 (6.4)
Unknown	11 (8.8)
Axillary node involvement	
N0	58 (46.4)
N1	23 (18.4)
N2	21 (16.8)
N3	20 (16.0)
Unknown	3 (2.4)
TNM Stage	
I	25 (20.0)
II	49 (39.2)
III	37 (29.6)
IV	7 (5.6)
Unknown	7 (5.6)
Family history^a^	
Yes	11 (8.8)
No	114 (91.2)
Sites of metastases at recurrence	
Lung	49 (39.2)
Bone	34 (27.2)
Liver	18 (14.4)
Brain	11 (8.8)

a Family history was defined as ≥1 first‐ or second‐degree relative with breast cancer at age ≤50 years or ≥1 close blood relative with epithelial ovarian cancer at any age.

### Germline mutations of 55 genes that affect BC susceptibility

All coding regions of 55 genes that affect BC susceptibility were sequenced in 26 patients with TNBC. Gene‐based case–control association analysis using Asian population information obtained from the 1000 Genomes Project database. Only deleterious mutations were included in this study. Of all 26 patients with TNBC 6 (23.1%) carried four different *BRCA1* mutations, three (11.5%) carried three different *PALB2* mutations, three (11.5%) carried three different *BRCA2* mutations, and the other three (11.5%) carried three different *CDH1* mutations. Notably, four patients carried the same *CHEK2* mutation (c.1246A>G). Deleterious mutation sites for each BC‐related gene detected in these patients are listed in Table S2.

For each discovery screen, genes with mutations in at least two patients are shown in Table 2. One heterozygous *BRCA1* frameshift mutation (rs80350973) was identified in three (11.5%) patients. Two heterozygous missense mutations—*CHEK2* (c.1246A>G) and *BRIP1* (c.587A>G)—were identified in 4 (15.4%) patients and 2 (7.7%) patients, respectively. One heterozygous in‐frame *BARD1* deletion mutation (c.1075_1095del) was identified in two (7.7%) patients. Details of these recurrent mutations are shown in Table 2.

**Table 2:** Detail information of recurrent mutations in 26 TNBC patients

Gene (Version)	Exon	Nucleotide change	Amino acid change	Type of mutation	dbSNP	PolyPhen prediction^a^	Clinical significance	Number of TNBC cases, *n* = 26	Allele frequency in Asian^b^
*BRCA1* (NM_007300.3)	24	c.5533_5540del ATTGGGCA	p.Ile1845Aspfs Ter3	frameshift variant (heterozygous)	rs80357973	N/A	unknown	3 (11.5%)	0.00%
*BARD1* (NM_000465.2)	4	c.1075_1095del TTGCCTGAATGTTCTTCACCA	p.Leu359_Pro365delinsdel	inframe deletion (heterozygous)	N/A	N/A	neutral polymorphisms 23	2 (7.7%)	0.00%
*BRIP1* (NM_032043.2)	4	c.587A>G	p.Asn196Ser	missense variant (heterozygous)	rs550707862	benign (0.004)	unknown	2 (7.7%)	0.00%
*CHEK2* (NM_001005735.1)	11	c.1246A>G	p.Lys416Glu	missense variant (heterozygous)	rs142470496	probably damaging (0.907)	unknown	4 (15.4%)	0.00%

a PolyPhen was used to predicts possible impact of single amino acid substitution on the structure and function of a human protein using straightforward physical and comparative considerations.

b The allele frequency of certain recurrent mutations in Asian dependent on the publication data from 1000 Genomes Project data.

N/A, Not applicable; TNBC, Triple‐negative breast cancer.

### Validation of germline mutation rs80350973

The rs80350973 mutation was further validated in an additional 100 TNBC patients using PCR and Sanger sequencing. In total, aside from a failed Sanger sequencing as a result of an unqualified DNA sample, this *BRCA1* frameshift mutation was detected in 9 (7.2%) of 125 TNBC patients.

### Clinical relevance of the rs80350973 mutation

According to the ClinVar database (https://www.ncbi.nlm.nih.gov/clinvar/variation/55591/) and Breast Cancer Information Core (BIC) (https://research.nhgri.nih.gov/projects/bic/Member/cgi-bin/bic_query_result.cgi?table=brca1_exons&nt=5589&base_change=del%20ATTGGGCA), the rs80350973 mutation was predicted to cause both a frameshift variant and a noncoding variant. As this frameshift mutation occurs in exon 24, it likely disrupts the second BRCT domain of BRCA1 protein (Fig. S2) which affects DNA damage repair and cell‐cycle checkpoint.

**Figure 1:**
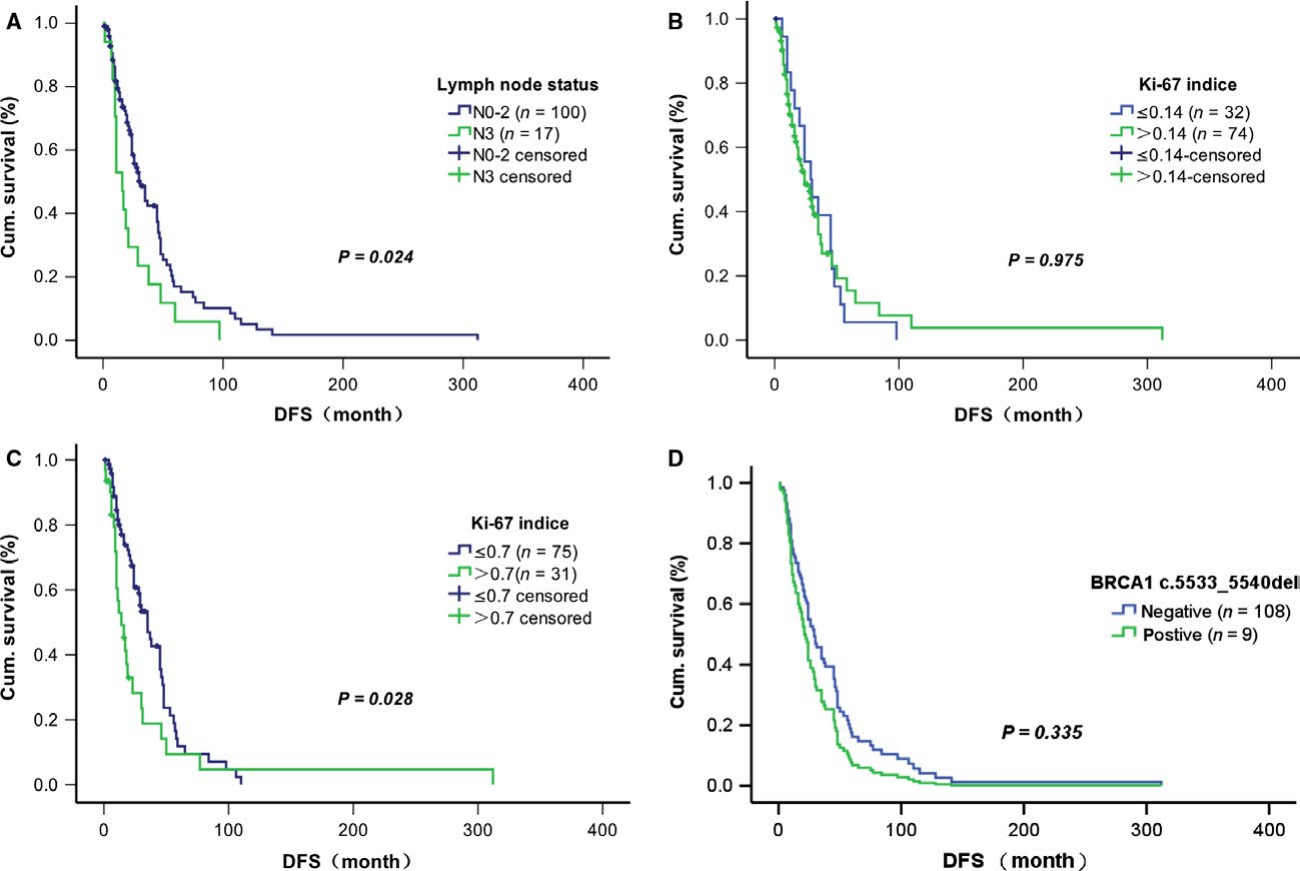
Effects of various factors on disease‐free survival among Chinese patients with Triple‐negative breast cancer. (A) Kaplan–Meier curves of lymph node status (N0–2 vs. N3). (B) Kaplan–Meier curves of Ki‐67 index at ≤0.14 versus >0.14. (C) Kaplan–Meier curves of Ki‐67 index at ≤0.7 versus >0.7. (D) Cox regression estimates by *BRCA1* rs80350973 mutational status.

Clinicopathological features of the 125 TNBC patients with or without the rs80350973 mutation are shown in Table 3. The rs80350973 mutation carriers and noncarriers did not significantly differ with regard to age at diagnosis, histological type, tumor size, lymph node status, TNM stage, family history, and visceral metastasis status at relapse. However, a higher Ki‐67 index was seen in rs80350973 mutation carriers than in noncarriers (*P *=* *0.004, Table 3). Kaplan–Meier survival analysis showed that patients with N3 stage lymph node status had significantly shorter DFS than did those with N0–2 status (*P *=* *0.024, Fig. 1A). Although the subgroups with high versus low Ki‐67 indices (≤0.14 vs. >0.14, as recommended by the St. Gallen International Expert Consensus of 2011) showed no statistical discrepancy (*P *=* *0.975, Fig. 1B), shorter DFS was seen in the Ki‐67 index >0.7 group than in the ≤0.7 group (*P *=* *0.028, Fig. 1C). Statistical discrepancies in DFS between rs80350973 mutation carriers and noncarriers were not observed with regard to other clinicopathological features. A covariate Cox regression model showed no statistical difference between rs80350973 mutation carriers and noncarriers with regard to DFS (*P *=* *0.335, Fig. 1D).

**Table 3:** Comparison of clinicopathological features between BRCA1 rs80350973 mutation carriers and noncarriers

Characteristic	Noncarriers (*n* = 116)	rs80350973 carriers (*n* = 9)	*P*‐value
Age at diagnosis (years)			
>35	102	7	0.382
≤35	14	2	
Histology of primary tumor			
Ductal	100	7	0.598
Lobular	8	0	
Others	7	1	
Primary tumor size			
T1	44	1	0.343
T2	45	6	
T3	9	1	
T4	7	1	
Axillary node involvement			
N0	51	7	0.253
N1	23	0	
N2	20	1	
N3	19	1	
TNM Stage			
I	24	1	0.426
II	43	6	
III	35	2	
IV	7	0	
Family history			
Yes	9	2	0.140
No	107	7	
Visceral metastases at relapse^a^			
Negative	61	6	0.415
Positive	55	3	
Ki‐67 index			
Median	0.48	0.75	**0.004**
Range	0.00~0.98	0.50~0.90	

a Patient with one or more metastatic sites including lung, liver, or brain at time of relapse was defined as visceral metastasis positive, otherwise, the patients were defined as negative.

## Discussion

Medical consultations for patients with newly diagnosed BCs increasingly use genetic counseling and testing. The National Comprehensive Cancer Network (NCCN) recently recommended *BRCA1/2* testing among women with BC diagnoses who met well‐established criteria 15. In 2010, the NCCN guideline suggested that *BRCA1/2* testing was indicated for women with TNBC who were younger than 40 years, based on emerging evidence that the triple‐negative phenotype was associated with hereditary cancer syndromes regardless of family history 16, 17. In 2013, the NCCN guidelines officially included women younger than 60 years with TNBC among those for whom genetic testing was appropriate 18.

Earlier studies with enormous cohorts have focused on the prevalence of *BRCA1/2* mutations in different patient subgroups classified by race, ethnicity or geographical factors 18, 19, 20, 21. Other TNBC susceptibility genes such as *BARD1* and *CHEK2* were also detected at relatively high frequency 22, 23 which concords with our findings (Table 2). However, little is known about recurrent germline mutations of *BRCA1/2* and its clinical significance in Chinese patients with TNBC. Aside from mutations at specific identifiable sites, their clinical interpretation may be challenging. In this study, we focused on a recurrent germline mutation of *BRCA1* and its clinical relevance in Chinese patients with TNBC. We observed that a frameshift *BRCA1* mutation (rs80350973) occurs at high frequency (9/125, 7.2%). This mutation was first reported by Suter et al. 24. in 2004, who identified it in two of 645 (0.3%) sporadic BC patients in Shanghai, China, but not in 319 unaffected health controls or in 342 patients with benign breast disease 24. The rs80350973 mutation was also identified in one of 60 (1.7%) Korean women with early‐onset BC (age ≤40 years) 25 and in one of 70 (1.4%) Chinese women with early‐onset BC (age ≤35 years) 26. In 2008, a larger cohort study of Chinese Han nationality was carried out by Li et al. 27., aimed at identifying of recurrent *BRCA1/2* mutations. They found the rs80350973 mutation in 4 of 489 (0.8%) women with family histories of BC and/or early‐onset BC. Among these four carriers, two were diagnosed when younger than 35 years, one of whom had a family history of gastric cancer; the other two carriers had family histories of BC. Two rs80350973 mutation carriers (0.5%) were also found among 426 sporadic BC cases; both were diagnosed when older than 35 years, and neither reported family histories of breast or ovary cancer 27.

According to the studies mentioned above, rs80350973 mutation prevalence remained at a low level among Chinese women with sporadic BC (0.3–0.5%), and showed no significant correlation with family cancer history or early age of onset.

Our results show, for the first time, the high prevalence (7.2%) of rs80350973 mutation among 125 Chinese women with TNBC, which indicates its prevalence in this molecular subtype of BC.

So far, studies have shown that this mutation, which is considered deleterious, is only found in Asian people 12, 27. However, its clinical relevance in TNBC remained unclear. According to ClinVar and BIC database, the rs80350973 mutation induces a frameshift mutation in exon 24 of the *BRCA1* gene, which could disrupt the BRCT domain (AA1784‐AA1863). This BRCT domain modulates interactions between BRCA1 and proteins that are phosphorylated in response to DNA damage 28; it has also been shown to bind directly to DNA double‐strand breaks (DSB) 29 and affects execution of the cell‐cycle checkpoint 30, 31. Because the BRCT domain could affect proliferation through the cellular checkpoint, disruption of this domain might lead to uncontrolled cell growth. Our data showed that the *BRCA1* rs80350973 mutation is only associated with high Ki‐67 index in Chinese patients with TNBC (*P *=* *0.004). A previous study showed that overexpression of Ki‐67 was associated with *BRCA1* mutation 32 and was an indicator of poor prognosis in TNBC 33, 34. Thus, we sought to determine if the rs80350973 mutation could contribute to shorter DFS. However, we failed to find a statistical difference in DFS between rs80350973 mutation carriers and noncarriers (*P *=* *0.335).

In conclusion, this study found a high mutational prevalence of rs80350973 in Chinese patients with TNBC, indicating a mutational prevalence of this variant. Bio‐informatics predictions further revealed that this mutation might disrupt the BRCT domain. As the BRCT domain critically affects DSB recognition and execution of checkpoint function, we supposed that rs80350973 mutation contributes to uncontrolled cell growth. This hypothesis is supported by our clinical result that rs80350973 mutation was more frequent in patients with higher Ki‐67 indices. Further functional studies to ascertain the molecular mechanisms behind rs80350973 mutation‐derived biological behaviors (such as cell proliferation and drug sensitivity) are warranted.

## Conflict of Interest

No potential conflicts of interest were disclosed.

## Supporting information


**Figure S1.** Identification of *BRCA1* rs80350973 mutation in 100 TNBCs using Sanger sequencing.

**Figure S2.** Location of rs80350973‐induced secondary structure variation in BRCA1 protein.

**Table S1.** Panel list of 55 genes that affect susceptibility to breast cancer.

**Table S2.** Exome sequencing of 55 genes that affect susceptibility to breast cancer in 26 patients with TNBC.
