# Supplementary figures and images for: Identification of recurrent BRCA1 mutation and its clinical relevance in Chinese Triple‐negative breast cancer cohort
Source: Cancer Med. 2017 Jan 30;6(3):547–54. doi: 10.1002/cam4.1004 (PMC5345662; doi:10.1002/cam4.1004)

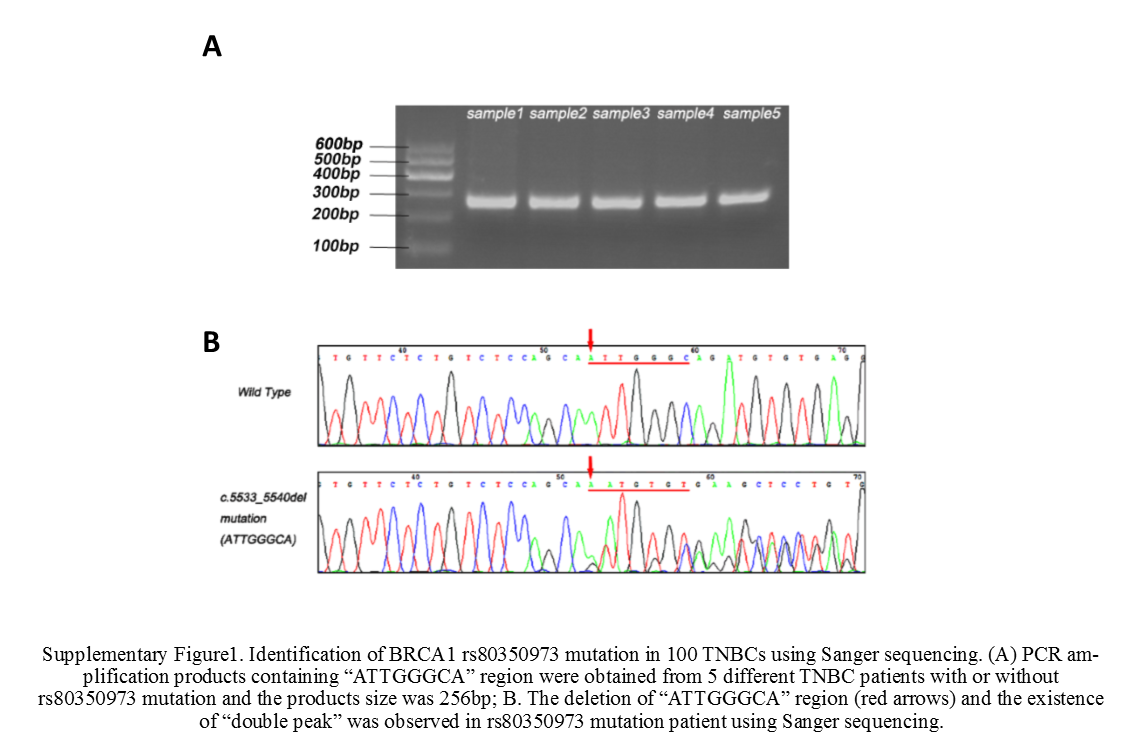

Supplement: Supplementary file 1 — Figure S1. Identification of BRCA1 rs80350973 mutation in 100 TNBCs using Sanger sequencing. [file CAM4-6-547-s001.tif]

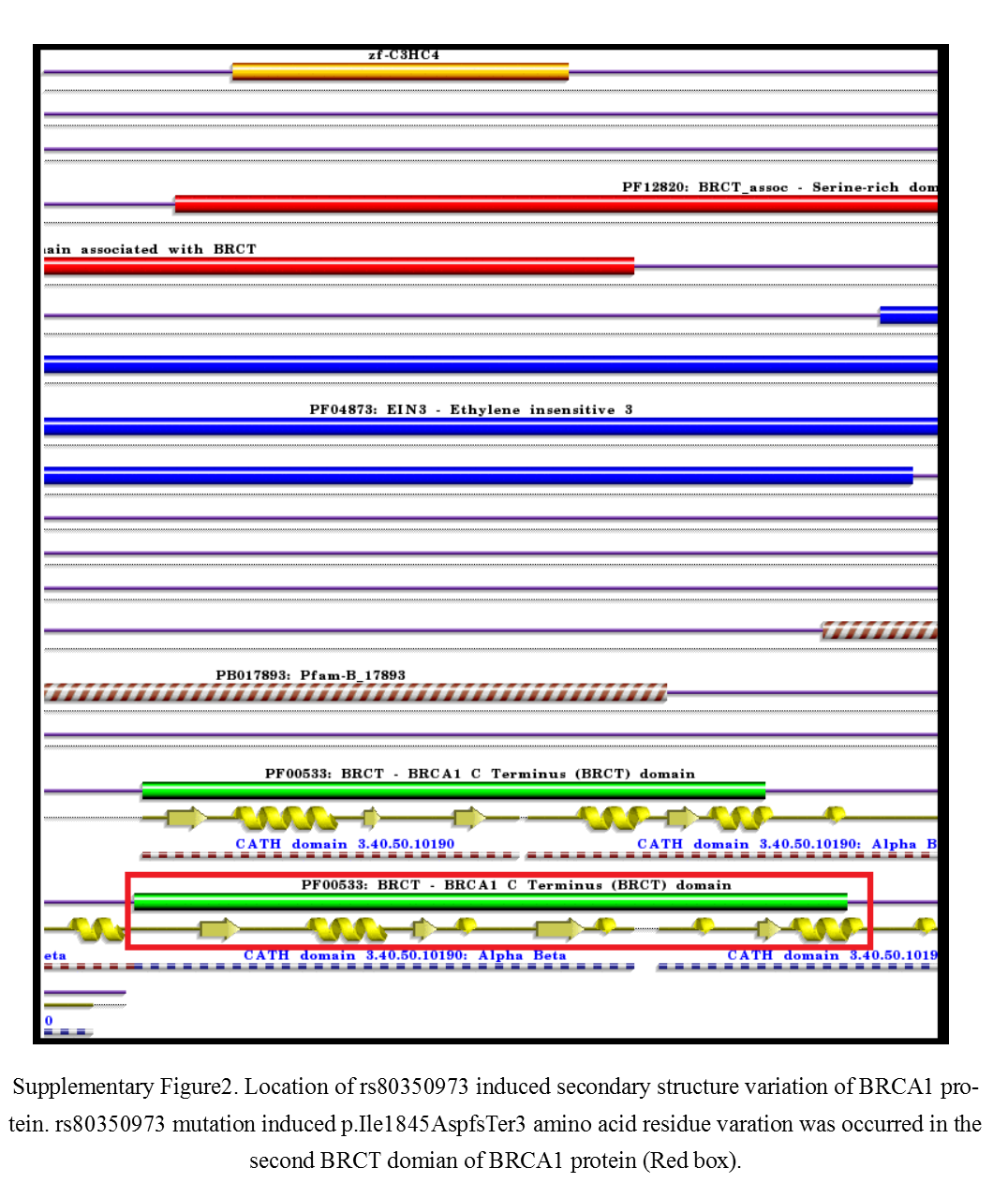

Supplement: Supplementary file 2 — Figure S2. Location of rs80350973‐induced secondary structure variation in BRCA1 protein. [file CAM4-6-547-s002.tif]
